# Dairy Products Are Not Adversely Associated with Depressive Symptoms over 6 Years in the Hispanic Community Health Study/Study of Latinos

**DOI:** 10.3390/nu18111805

**Published:** 2026-06-03

**Authors:** Anne Bodenrader, Daniela Sotres-Alvarez, Maria Carlota Dao, Tammy M. Scott, Semra A. Aytur, Sabrina E. Noel, Qibin Qi, Linda C. Gallo, Martha Daviglus, Wassim Tarraf, Robert Kaplan, Sherman J. Bigornia

**Affiliations:** 1 Department of Agriculture, Nutrition and Food Systems, University of New Hampshire, Durham, NH 03824, USA; anne.bodenrader@unh.edu (A.B.); carlota.dao@unh.edu (M.C.D.); 2 Department of Biostatistics, Gillings Global School of Public Health, University of North Carolina, Chapel Hill, NC 27599, USA; dsotres@email.unc.edu; 3 Friedman School of Nutrition and Science Policy, Tufts University, Boston, MA 02111, USA; tammy.scott@tufts.edu; 4 Department of Health Management and Policy, University of New Hampshire, Durham, NH 03824, USA; semra.aytur@unh.edu; 5 Department of Public Health, University of Massachusetts Lowell, Lowell, MA 01854, USA; sabrina_noel@uml.edu; 6 Department of Epidemiology and Population Health, Albert Einstein College of Medicine, Bronx, NY 10461, USA; qibin.qi@einsteinmed.edu (Q.Q.); robert.kaplan@einsteinmed.edu (R.K.); 7 Public Health Sciences Division, Fred Hutch Cancer Center, Seatle, WA 98109, USA; 8 Department of Psychology, San Diego State University, San Diego, CA 92182, USA; lgallo@mail.sdsu.edu; 9 Institute for Minority Health Research, College of Medicine, University of Illinois at Chicago, Chicago, IL 60612, USA; daviglus@uic.edu; 10 Institute of Gerontology & Department of Healthcare Sciences, Wayne State University, Detroit, MI 48202, USA; wassim.tarraf@wayne.edu

**Keywords:** dairy fat, depression, Hispanic/Latino, fermented dairy

## Abstract

**Background/Objectives**: Current evidence suggests that Hispanic/Latino adults experience a disproportionate burden of depression. Dairy consumption has been associated with fewer depressive symptoms, but examinations in Hispanic/Latino cohorts are unavailable. Our objective was to measure the 6-year prospective associations between dairy consumption and depressive symptoms among Hispanic/Latino adults. **Methods:** The Hispanic Community Health Study/Study of Latinos (HCHS/SOL) is a prospective population-based cohort study of 16,415 Hispanic/Latino adults residing in the US. We estimated daily dairy product consumption from two 24 h baseline dietary recalls using the National Cancer Institute method. The 10-item Center for Epidemiological Studies Depression Scale (CESD10) administered at baseline and follow-up assessed depressive symptoms. Survey multiple linear regression models adjusted for baseline CESD10 and other covariates, including sociodemographic, dietary and health factors. Standardized β coefficients represent the standard deviation difference in 6-year CESD10 score per one standard deviation increase in daily dairy intake at baseline. Complete data were available among 10,618 participants. **Results:** Neither baseline total dairy consumption (standardized β (95% CI); −0.019 (−0.048, 0.011)), nor milk (−0.006 (−0.029, 0.018)), cheese (0.038 (−0.006, 0.081)), or cream (−0.005 (−0.037, 0.028), *p* > 0.05 for all) consumption was significantly associated with the follow-up CESD10 score. Conversely, we observed a significant and inverse association between yogurt (−0.036 (−0.058, −0.013), *p* = 0.002) and butter (−0.049 (−0.092, −0.006), *p* = 0.027) with the CESD10 score. **Conclusions:** Total dairy, fat-based dairy groupings, milk, cheese, and cream were not associated with CESD10 score at 6-year follow-up; yogurt and butter showed inverse associations that require cautious interpretation due to very small effect sizes. Although additional prospective analyses in other diverse cohorts are needed to confirm these results, our findings suggest that dairy consumption is not adversely associated with depressive symptoms in Hispanic/Latino adults.

## 1. Introduction

In the US, an estimated increase of 18 million prevalent cases of depression occurred between 2015 and 2023 [1]. Moreover, depression is disproportionately experienced in the US. From 2017 to 2023, the rate of lifetime and current depression among Hispanic/Latino adults increased by 12.9% from 18.4% to 31.3%. Over the same period, it increased by 6.9% among non-Hispanic white adults from 22.3% to 29.0% [1]. Additionally, Hispanic/Latino adults experience nearly twice the risk of depressive symptoms relative to non-Hispanic white adults and experience greater severity of depression than other ethnic and minority groups [2]. While depression and depressive symptoms can stem from a range of sociological, economic, and psychological factors [3,4], an expanding body of evidence indicates that biological factors, including diet, may also play a role [5].

Meta-analyses of observational studies suggest that diets high in fruits, vegetables, and whole grains, and low in added sugars, are associated with a reduced incidence of depression and lower depressive symptoms [6,7]. In contrast, although dairy consumption is recommended as part of the US Dietary Guidelines for Americans [8], its potential role in depressive symptoms remains uncertain. Studies to date examining dairy products and depressive symptoms have reported mixed results [9,10,11,12,13,14,15,16,17,18,19,20,21,22].

Inconsistencies in the dairy–depressive symptoms literature may reflect heterogeneity in how dairy products are defined, which milk products are categorized as dairy, inadequate reporting of serving size, residual confounding from unmeasured dietary factors (e.g., total energy), and a lack of distinction between whole-fat and low-fat dairy products [9]. Most existing studies have relied on cross-sectional designs [11,13,14,15,16,17], which limit causal inference and may obscure associations. Furthermore, relatively few prospective cohort studies have investigated dairy intake in relation to depression or depressive symptoms in general population cohorts [18,22].

Chronic inflammation and cardiometabolic dysfunction are pathways implicated in depressive symptoms and depression [23,24]. These biological mechanisms may be particularly relevant to Hispanic/Latino adults who experience disproportionately higher rates of obesity [25] and the metabolic syndrome [26], conditions that elevate inflammation and cardiovascular burden. It has been reported in Hispanic/Latino cohorts that higher inflammatory cytokine concentrations are associated with greater depressive symptoms [27,28].

Indeed, evidence from clinical studies supports that dairy consumption beneficially impacts inflammation [29,30]. Dairy products may, in part, be associated with lower depressive symptoms because they are rich in anti-inflammatory nutrients, including vitamins A and D, magnesium [31], casein, and whey [32]. Moreover, dairy products, including butter and cream [33], are a source of short-chain [34], medium-chain [35], and odd-chain saturated fatty acids [36], as well as ruminant trans-fatty acids, which have also been beneficially associated with inflammatory burden.

While the underlying biological processes linking diet and mental health are likely universal, Hispanic/Latino adults represent a high-priority population for diet–depression research due to their disproportionate burden of obesity and metabolic syndrome. Because these conditions are characterized by chronic systemic inflammation, a known pathway in the development of depressive symptoms, this cohort provides an optimal setting to examine whether dietary factors with hypothesized anti-inflammatory properties, such as dairy and dairy fat, are associated with prospective depressive symptom outcomes. However, few prospective cohort studies exist that examine the potential impacts of dairy consumption, particularly those products with fat (full- and reduced-fat), on depressive symptoms, and none have examined these associations in Hispanic/Latino populations. To address current knowledge gaps, the objective of the current study is to quantify the prospective associations between baseline total dairy, whole and reduced fat dairy, nonfat dairy, and dairy product consumption (milk, cheese, yogurt, cream, and butter) and depressive symptoms measured 6 years later, adjusting for baseline depressive symptoms among Hispanic/Latino adults residing in the US. In order to fully examine the potential anti-inflammatory benefits of dairy fat, we included butter and cream in our definition of dairy, which have been historically excluded from dairy recommendations [37,38] specifically due to concerns regarding the fat content [37,38]. We hypothesized that dairy consumption would be inversely associated with depressive symptoms and that these associations would be stronger for full- and reduced-fat dairy products as compared to nonfat varieties.

## 2. Materials and Methods

### 2.1. Participants

The Hispanic Community Health Study/Study of Latinos (HCHS/SOL) is a longitudinal epidemiological study that surveyed multiple health parameters in 16,415 Hispanic/Latino US adults. Participants, aged 18–74 years, lived in areas surrounding four high-density Hispanic/Latino communities within Miami, FL, San Diego, CA, Chicago, IL, and Bronx, NY. They were targeted for sampling based on census blocks cross-classifying populations by high and low Hispanic/Latino concentration and high and low educational status from the 2000 US decennial census. Participants self-identified as Hispanic/Latino and were classified according to their personal or family place of origin as: Mexico, Cuba, Puerto Rico, Dominican Republic, and Central and South America. Baseline data collection was conducted between 2008 and 2011, with annual telephone interviews conducted to identify cardiovascular and pulmonary events. A follow-up clinic examination was conducted approximately 6 years later (2014–2017). Exclusion criteria included anyone planning to move away in the next 3 years and anyone unable to provide informed consent or attend clinical evaluations due to health conditions, disabilities, or mental problems [39,40].

Of the 16,415 participants at baseline, 11,623 returned for the second clinic visit. Of those, 603 were excluded due to missing CESD10 score at baseline or visit, 2126 because of missing dietary information (exposure), and an additional 260 because of other missing covariates. In total, 10,618 had complete data available for analysis. Participants provided witnessed and formally recorded informed consent. Surveys were interview-administered in participants’ preferred language. This study was conducted according to guidelines established in the Declaration of Helsinki, and all HCHS/SOL procedures were approved by the Institutional Review Boards at all participating institutions (coordinating center: University of North Carolina at Chapel Hill, Chapel Hill, NC; field centers: Albert Einstein College of Medicine, Bronx, NY, San Diego State University, San Diego, CA, University of Illinois at Chicago, Chicago, IL, and University of Miami, Miami, FL) and are currently governed by a single IRB at University of North Carolina at Chapel Hill, Chapel Hill, NC (07-1003, 22 October 2025) [41].

### 2.2. Dietary Assessment and Dairy Consumption

Diet was assessed at baseline using two 24 h dietary recalls administered by bilingual, centrally trained registered dietitians. The first was obtained in person at the initial baseline visit and the second between 5 and 30 days later via telephone. Dietary assessment was guided by the Nutrition Coordinating Center at the University of Minnesota, School of Public Health (NCC) (Minneapolis, MN). The Diet and Supplement Subcommittee of the HCHS/SOL worked to identify Hispanic/Latino foods and recipes to add to the NCC’s Nutrition Data System for Research (NDSR (version 11)) Food and Nutrition Database [42].

The nutrient content of reported foods was analyzed using the NDSR database. The NDSR database includes more than 19,000 foods [43], allowing for estimation of total dairy intake, including from complex foods like cheese on pizza or used in recipes, such as butter and milk for cakes or puddings. We assessed intake of all dairy products, including milk, cheese, yogurt, butter, and cream, by fat level from all sources, including from complex foods and recipes. Servings were calculated for each 24 h recall and categorized into either milk, cheese, yogurt, cream, or butter and grouped by fat content into whole- and reduced-fat dairy intake or nonfat dairy intake.

Consistent with previous HCHS/SOL studies of diet, the National Cancer Institute (NCI) method was used to estimate the serving amount of dairy products consumed from the available 24 h dietary recalls [42,44]. Using dietary recalls to estimate a food or nutrient’s association with an outcome is inherently vulnerable to measurement error due to self-report bias and intra- and inter-person variability. The NCI method addresses variability in dietary recalls by using the complete cohort to calculate the probability of a given food’s consumption on any day in conjunction with the average amount a specific person consumes on a day when that food is eaten. This statistical method helps account for day-to-day variation and provides predicted usual intake estimates for each member of the cohort based on model covariates and recall data [42,44]. Data from individuals with either 1 or 2 available 24 h recalls were analyzed using the 2-part NCI model, adjusting for age, sex, Hispanic/Latino background (country of origin), and field center. Consistent with HCHS/SOL methodology, dietary recalls with extreme values (sequence-gender-specific observed daily energy intake (kcal/d) below the first and above the ninety-ninth percentiles) or with unreliable data (designated for recalls where participants were unable to recall more than one meal or were flagged as unreliable for other reasons during review by dietary and lead interviewers) were discarded.

Total dairy was calculated as the sum of servings per day (s/d) from milk, cheese, yogurt, cream and butter. Cream and butter were included in the total dairy variable, as they are sources of dairy fats, some of which have been shown to have anti-inflammatory properties. Full and reduced fat dairy included whole and reduced fat milk, cheese, yogurt, cream and butter. Serving sizes were defined using the recommended serving size from the 2000 Dietary Guidelines for Americans, when available, or from the recommended serving size from the Food and Drug Administration for foods not included in the Dietary Guidelines [37,45]. Serving sizes were defined as 1 cup for milk and yogurt, 1.5 ounces of natural cheese or 2 ounces of processed cheese, 1 tablespoon of cream, and 1 teaspoon of butter.

The primary dairy variables were total dairy, full and reduced fat dairy, nonfat dairy, and individual dairy products (milk, cheese, yogurt, cream, and butter) in s/d. In exploratory analyses we examined total dairy as that from milk, cheese, and yogurt only, as defined in the 2000 Dietary Guidelines for Americans and the US Department of Agriculture’s MyPlate guideline [37,45].

### 2.3. Assessment of Depressive Symptoms

Depressive symptoms were assessed at both visits using the 10-item Center for Epidemiological Studies Depression Scale (CESD10) [46], which has been validated for use in Hispanic/Latino populations [47]. The CESD10 is a survey of how often a participant has been experiencing depressive symptoms over the previous week. Responses range from “Rarely or none of the time (less than 1 day)”, which is scored as a 0, to “Most or all of the time (5–7 days)”, which scores 3. Two positively worded items (“I felt hopeful about the future” and “I was happy”) were reverse scored. Total scores for the CESD10 range from 0 to 30, where higher scores indicate more depressive symptoms [47]. The primary outcome was the CESD10 score from visit 2 on a continuous scale.

### 2.4. Covariates

Covariates measured at baseline were age, sex, field center, Hispanic/Latino background, education level, income level, acculturation, smoking status, CESD10 score, lapsed time between visits in days, body mass index (BMI), physical activity level expressed in metabolic equivalents, use of antidepressants, history of cardiovascular disease (CVD), hypertension, type 2 diabetes (T2D), and total energy. Additional dietary covariates were added sugar (g/d), omega-3 fatty acids (mg/d), whole grains (s/d), fruits (s/d), and vegetables (s/d).

History of CVD defined by the Framingham Study criterion [48] was based on 12 self-reported questions ascertaining medical history of coronary heart disease, cerebrovascular disease, or peripheral arterial disease. Clinical measures included ankle–brachial index, intermittent claudication in either leg, or electrocardiogram results to establish a history of CVD. Any affirmative answer to any of the 12 questions or abnormal clinical measures was coded as 1; otherwise, as 0. This variable was designed to capture positive cases of CVD; as such, participants with incomplete information were coded as 0 (no/unknown history of CVD) [48].

History or presence of hypertension was defined by the National Health and Nutrition Examination Survey definition of blood pressure greater than or equal to 140/90 mm/Hg or self-reported use of antihypertensive medications [48]. Participants meeting the definition for hypertension were given an indicator score of 1; participants missing clinical values who were not taking antihypertensive medication and did not report a diagnosis of hypertension were assumed to be not hypertensive.

Diabetes was assessed by self-report, use of hypoglycemic medications, or the American Diabetes Association criteria for T2D [48]: a fasting blood glucose of greater than or equal to 126 mg/dL, a 2 h oral glucose tolerance test greater than or equal to 200 mg/dL, or a glycosylated hemoglobin A1c greater than or equal to 6.5%. Participants meeting the criteria for T2D were given a 1; participants with missing clinical values who were not using hypoglycemic medications and did not report a diagnosis of diabetes were assumed to be non-diabetic.

Physical activity level was assessed using the World Health Organization Global Physical Activity Questionnaire (GPAQ) [49]. The GPAQ surveys participants on their activity levels in three areas of their lives: work, transportation and recreation. For each area, participants answer a variety of questions to quantify the amount of time spent on various activities and their activity level (vigorous or moderate). The GPAQ yields a continuous variable that estimates an individual’s average daily energy expenditure. One metabolic equivalent (MET) is defined as 1 kcal/kg/hour and is equivalent to the energy cost of sitting quietly for one hour [48].

Some dietary factors are associated with inflammation and may confound associations between dairy consumption and depressive symptoms. As such, models included total energy (kcals/d) consumption, as well as the intakes of added sugar (g/d), omega-3 fatty acids (mg/d), whole grains (s/d), and fruit (s/d) and vegetables (s/d) as covariates.

### 2.5. Statistical Analyses

Data were analyzed using SAS version 9.4. All continuous variables were examined for normal distribution. Survey multiple linear regression (PROC SURVEYREG in SAS) was used to conduct the analyses to account for the complex population-based sampling method [40]. Baseline and visit 2 CESD10 scores were skewed. We conducted two sets of regression analyses, one using Box–Cox-transformed CESD10 scores and another using the untransformed scores. Both approaches yielded comparable results. To improve the interpretability of our findings, we present the analyses using the untransformed CESD10 scores. Sample characteristics are presented as mean (95% CI) or proportions, and regression results as β (95% CI). To foster the comparison of estimated effect sizes between dairy products, standardized β estimates are also reported. Significance was determined at *p* ≤ 0.05.

To examine the impact of baseline dairy consumption on CESD10 score at visit 2, we fit the following models: Model 1 adjusted for baseline CESD10 score as well as baseline socio-demographic and clinical confounders, including age, sex, field center, Hispanic/Latino background, education level, income level, acculturation, smoking status, lapsed time between visits in days, BMI, physical activity level, use of antidepressants, history of CVD, hypertension, T2D, and total energy. Model 2 included dietary confounders previously shown to correlate with depressive symptoms or depression. Covariates were Model 1 covariates plus added sugar (g/d), omega-3 fatty acids (mg/d), whole grains (s/d), fruits (s/d), and vegetables (s/d). Adjustment for multiple testing was not conducted because the primary analyses were prespecified and focused on dairy exposures in relation to a single outcome. Also, multiple-testing adjustment may be overly conservative and can increase type II errors [50,51]. Post hoc power analyses using observed effect sizes and confidence limits at α = 0.05 were conducted using G*power (Version 3.1.9.7).

In exploratory analyses, we examined total dairy defined as total milk, cheese, and yogurt (s/d) (omitting cream and butter). In further sensitivity analyses, we examined all exposures and outcomes excluding participants who used antidepressant medications at baseline (*n* = 1041) to mitigate confounding effects due to a previous diagnosis of depression. The modeling structure for these analyses was similar to that used for the primary analysis, except that antidepressant medication use was removed as a covariate for analyses excluding users of antidepressant medications. Lastly, we compared baseline CESD10 scores between participants in the current analytical sample (*n* = 10,618) and those who did not return for the follow-up visit (*n* = 4792) in age-, sex-, and field-center-adjusted analyses.

## 3. Results

### 3.1. Descriptive Characteristics

Sociodemographic and clinical characteristics at baseline are shown in Table 1. The mean baseline age was 43.4 y (95% CI: 42.8 y, 43.9 y), and the majority were female (56.1%) (Table 1).

The average estimated intakes (s/d) of total dairy, milk, cheese, yogurt, cream, and butter were 3.02 s/d (95% CI: 3.00, 3.04); 0.82 s/d (0.81, 0.83); 0.50 s/d (0.50, 0.51); 0.10 s/d (0.09, 0.10); 0.89 s/d (0.89, 0.90); and 0.71 s/d (0.70, 0.72), respectively (Table 2).

### 3.2. Associations Between Dairy and Dairy Product Consumption and Depressive Symptoms

The average CESD10 score at the baseline and visit 2 were 7.30 (95% CI: 7.10, 7.51) and 6.56 (6.34, 6.78), respectively. Although total, full- and reduced-fat, and nonfat dairy products were inversely associated with the visit 2 CESD10 score, these associations were not statistically significant (Table 3, *p* = 0.22 to 0.46, Model 2). We observed null associations of milk, cheese, and cream consumption with CESD10 score (Table 4, *p* = 0.09 to 0.78, Model 2). Conversely, each additional daily serving of yogurt at baseline was associated with a 4.2-point lower CESD10 score at visit 2 (95% CI: –6.83, –1.56; *p* = 0.002; Model 2). Likewise, each additional daily serving of butter at baseline was associated with a 1.08-point lower CESD10 score (95% CI: –2.04, –0.12; *p* = 0.027; Model 2). Post hoc power estimates were 21% for total dairy, 17% for full- and reduced-fat dairy, 11% for nonfat dairy, 8% for total milk, 40% for total cheese, 88% for total yogurt, 6% for total cream, and 60% for butter.

### 3.3. Additional Analyses

A modified total dairy product consumption variable, defined as milk, cheese, and yogurt only (i.e., excluding cream and butter), was not associated with CESD10 score (Appendix A, Table A1, *p* = 0.46 to 0.95, Model 2). In analyses excluding 1041 individuals taking antidepressant medication at baseline, associations remained consistent with the primary analysis (Appendix A, Table A2, Model 1: β = −0.23, 95% CI: −0.56, 0.10, *p* = 0.18; Model 2: β = −0.24, 95% CI: −0.57, 0.09, *p* = 0.15). Total Yogurt and Total Butter continued to show small but significant effect sizes in Model 1 (Yogurt: β = −3.75, 95% CI: −6.33, −1.17, *p* = 0.004; Butter: β = −1.49, 95% CI: −2.44, −0.54, *p* = 0.002) and Model 2 (Yogurt: β = −3.69, 95% CI: −6.28, −1.11, *p* = 0.005; Butter: β = −1.44, 95% CI: −2.40, −0.47, *p* = 0.004). Participants in the current analytical sample (*n* = 10,618) had a mean baseline CESD10 score of 7.27 (95% CI: 7.08, 7.47), which was not significantly different from the mean score of 7.42 (95% CI: 7.18, 7.66) observed among individuals lost to follow-up (*p* = 0.30).

## 4. Discussion

In this study of Hispanic/Latino adults residing in the US, we observed a null association between baseline total dairy consumption and depressive symptoms measured at 6 years’ follow-up. These results remained the same when investigating fat product level (whole and reduced fat and nonfat), as well as separately for milk, cheese, and cream. We observed that higher yogurt and butter intakes were significantly associated with fewer depressive symptoms 6 years later, approximately equivalent to moving from non-consumption to consumption. However, the size of the standardized beta estimates for yogurt and butter was very small and similar in absolute size to other dairy products where null associations were observed. Collectively, our results suggest that dairy consumption may not be adversely associated with depressive symptoms among Hispanic/Latino adults.

### 4.1. Total Dairy Intake and Depressive Symptoms

To date, the associations of total dairy consumption and depressive symptoms or depression have not been widely investigated [9,11,14,15,16,17,18,22,52]. Similar to the present analysis, only two prior studies employed a prospective cohort design [18,22]. In a recent study of Finnish men (42 to 60 years of age), baseline total dairy intake (grams/d), including milk, cheese, yogurt, cream and ice cream, was not associated with incidence of depression over 24 years of follow-up [22]. Interestingly, total fermented dairy (excluding cheese) was inversely associated with depression risk, while non-fermented dairy showed an adverse association. Although we did not distinguish between fermented and non-fermented dairy, we observed a beneficial association between total yogurt consumption and depressive symptoms. In contrast to the current study, the Finnish cohort analysis did not account for dairy consumed in mixed dishes (e.g., pizza or burgers) or express intakes in servings. Both may lead to misclassification of dairy intake due to variation in accompanying foods and differences in dairy product density.

Another prospective cohort study of Taiwanese adults (≥65 years of age) examined the frequency of consumption of multiple food groups, including total dairy, in relation to 4-year depressive symptoms [18]. Consistent with our findings, higher baseline dairy frequency was not associated with depressive symptoms. However, in that study, residual confounding by other dietary factors like total energy and vegetables likely biased effect estimates. Moreover, dairy was expressed in consumption frequencies (times/week) rather than grams or servings, which can contribute to misclassification of dairy intake. Our study builds on these prior investigations, minimizing misclassification of dairy through extraction of dairy from mixed dishes and standardizing intakes in servings.

Additionally, cultural patterns of dairy consumption substantially differ across populations. In Japanese and other Asian cohorts, dairy intake is relatively low and consists mainly of milk, whereas in many European populations dairy contributes a larger share of total energy intake and includes a wider variety of products such as yogurt and cheese [53]. Because our study focuses on Hispanic/Latino adults living in US urban centers, direct comparisons with these populations may be limited.

### 4.2. Individual Dairy Products and Depressive Symptoms

The associations of individual dairy products with depressive symptoms have been examined in a limited number of cross-sectional studies, including those on milk [13,14,15,52], yogurt [12,13,14,15], cheese [14,15], and cream [14]; butter as an individual dairy product has generally not been assessed [14,15,16,17].

One recent prospective study examined the association between milk consumption by fat level and depression 8 years later in the UK Biobank cohort (mean age 56 years). Similar to our study, the investigators found a null association between total milk consumption and depression despite using different measurement methods for dietary intake (FFQ) and depression (items 1 and 2 on the Patient Health Questionnaire 4 screening tool) [19].

To our knowledge, only one prospective cohort study examined yogurt specifically [21]. Using data from the SUN Cohort Study of Spanish university alumni (mean age 38 years), higher consumption of whole-fat yogurt, but not low-fat, was associated with reduced 10-year incidence of depression [21]. Our findings for yogurt are consistent with this result and with randomized controlled trials demonstrating that probiotic supplementation can reduce depressive symptoms in individuals both with and without major depressive disorder [54].

### 4.3. Biological Plausability: Yogurt and Butter

We observed that yogurt and butter consumption were significantly and inversely associated with CESD10 scores. It is expected that the effect size of any single food on a health outcome will be small. However, the standardized β estimates for yogurt and butter (0.036 and 0.049) fall below the suggested conventional threshold value of 0.10 for a small effect [55]. These effect sizes were also comparable to those for milk, cheese, and cream (ranging from 0.0045 to 0.038). This pattern was reflected in the low post hoc power estimates observed for most analyses, which were largely driven by the small observed effect sizes, wide confidence limits, and corresponding *p* values [56,57]. Consequently, these results should be interpreted with caution, as they may be indistinguishable from the measurement errors inherent to self-reported dietary assessment and the CESD10 scale, and thus may not be clinically relevant.

Despite these very small effect sizes, several biological mechanisms may support a protective role for yogurt. As a fermented product rich in probiotics [58], yogurt may increase SCFA levels through direct ingestion or stimulation of the gut microbiome [59,60]. Higher dietary intake and circulating levels of SCFAs have been associated with reduced inflammation and improved insulin sensitivity [61,62]. In addition, probiotics can produce or interact with neurotransmitters like GABA, serotonin, dopamine, and glutamate, potentially influencing mental health [10,63,64].

Regarding butter, while historical evidence emphasizes moderating saturated fat for cardiovascular health [65], more recent meta-analyses of long-term cohort studies suggest null associations between butter consumption and cardiovascular disease [66] and all-cause mortality [66,67]. Butter is a source of ruminant trans-fatty acids, including vaccenic and conjugated linoleic acids [68,69], which may exert anti-inflammatory effects [33] through activation of peroxisome proliferator-activated receptors [33]. Given the lack of prior epidemiological studies on butter and depression or depressive symptoms, any benefit butter may confer requires further clarification in future research.

### 4.4. Strengths

The current study has several notable strengths. It is the first study to look at dairy and depressive symptoms in a Hispanic/Latino cohort, using data from a large and diverse sample of 10,618 Hispanic/Latino adults. The use of a prospective design with 6 years of follow-up reduced the possibility for reverse causation. Only two other studies examining dairy and depressive symptoms have used a prospective design. Our estimates of dairy consumption included dairy as a component of mixed dishes where dairy is an ingredient (e.g., cream-based soups), which reduced the potential for non-differential misclassification bias. We also examined the associations of dairy by fat level, which has not been done in other prospective studies of total dairy. We adjusted for multiple possible confounders, including other dietary factors that previous studies in this area did not consider, such as total energy.

### 4.5. Limitations

There are, however, limitations to consider. Diet was ascertained by a 24 h recall, which is subject to nondifferential misclassification of dietary intakes. This issue could have contributed to observed null associations. However, we used the NCI method, which has been validated to estimate usual intakes of nutrients and episodically consumed foods [44,70,71]. Diet was assessed at the baseline visit only. Although diet has been shown to remain stable in longitudinal cohort studies of middle-aged adults [72,73], it is possible that dairy intake changed during the follow-up period. Future studies with repeated measures of dairy intake (self-reported and biomarkers) are needed to confirm our findings. Despite adjustment for multiple confounders, residual confounding remains a concern. For example, we were unable to assess potential confounding due to lactose intolerance, access to mental health care, and psychosocial stressors either because the data was not collected or was only available in a subset of participants. There was substantial loss to follow-up, which could contribute to a biased sample. While it has been previously reported that participants lost to follow-up tended to be males, unmarried, younger, have less than a high school education, and were born in the US [74], other potentially important differences may still exist. It is also noteworthy that some Hispanic/Latino people may be uncomfortable acknowledging depressive symptoms due to cultural stigmatization, which may have caused underreporting of depressive symptoms in our population, contributing to attenuated effect sizes [75]. Lastly, we were not able to use clinically diagnosed depression as an outcome.

## 5. Conclusions

In conclusion, consumption of total dairy, full- and reduced-fat dairy, and nonfat dairy, as well as milk, cheese, and cream, were not associated with depressive symptoms (CESD10) measured 6 years later in HCHS/SOL, the largest epidemiological study of diverse Hispanic/Latino adults in the US. Yogurt and butter consumption showed small inverse associations that require cautious interpretation and replication in future studies. Our study contributes to the understanding of the associations between dairy consumption and depressive symptoms by using a prospective design and extending the current evidence base to include Hispanic/Latino adults. Although an analysis of a single population cannot determine if these associations are unique to Hispanic/Latino adults compared to other groups, our findings provide critical prospective data for a community disproportionately affected by cardiometabolic and inflammatory risk factors. While additional prospective analyses in other diverse cohorts are needed to confirm these results, our findings suggest that dairy consumption is not adversely associated with depressive symptoms in Hispanic/Latino adults.

## Figures and Tables

**Table 1:** Baseline demographic and descriptive characteristics by quintile of dairy servings ^1^, HCHS/SOL.

Characteristics	Q1 *n* = 2123	Q2 *n* = 2124	Q3 *n* = 2124	Q4 *n* = 2124	Q5 *n* = 2123	Overall *n* = 10,618
Age, years	44.46 (43.52, 45.39)	42.79 (41.75, 43.84)	43.73 (42.74, 44.73)	43.16 (42.13, 44.19)	42.67 (41.55, 43.79)	43.35 (42.77, 43.94)
Body Mass Index, kg/m^2^	29.78 (29.39, 30.18)	29.57 (29.25, 29.89)	29.45 (29.03, 29.88)	29.34 (28.98, 29.70)	29.62 (29.21, 30.03)	29.75 (29.56, 29.95)
Physical Activity, MET-min/d ^2^	711.3 (646.0, 776.6)	608.8 (544.1, 673.4)	607.6 (549.0, 666.2)	709.4 (636.8, 781.9)	674.1 (612.6, 735.7)	574.6 (548.1, 601.1)
Female, %	52.4	60.4	64.4	58.0	46.8	56.1
High School Educational Attainment, %	62.7	66.8	67.4	68.7	72.7	68.1
Current Smoker, %	21.1	18.5	17.6	16.6	21.0	18.9
Cardiovascular Disease, % ^3^	21.9	21.7	23.7	24.7	26.2	23.9
Hypertension, % ^4^	25.4	25.9	27.9	25.2	28.6	26.7
Type 2 Diabetes, % ^5^	18.4	15.0	16.5	16.0	15.2	16.1
Medical Antidepressant Use, %	4.1	5.2	7.0	5.9	5.9	5.7
Born in the United States, %	14.9	17.5	15.2	20.8	28.2	20.0
Background						
Central American, %	17.0	9.2	6.3	3.9	3.6	7.3
Cuban, %	17.9	20.1	22.1	22.1	19.4	20.4
Dominican, %	4.6	6.8	9.2	13.2	8.7	8.8
Mexican, %	45.9	46.1	44.7	38.2	29.7	40.1
Puerto Rican, %	7.8	8.5	10.1	13.1	29.0	14.7
South American, %	4.8	6.3	5.1	5.4	4.4	5.2
Other, %	1.9	3.1	2.5	4.1	5.1	3.5
Center						
Bronx, %	3.8	9.8	20.1	33.7	47.9	25.4
Chicago, %	45.4	25.5	14.8	8.2	2.4	17.1
Miami, %	37.4	33.7	31.7	27.2	21.6	29.5
San Diego, %	13.5	31.0	33.4	30.9	28.0	28.0

^1^ Data are mean (95% CI) or proportion values by dairy consumption category. Participants were categorized by quintile of total dairy intake after adjusting dairy intake for total energy using the residual method. ^2^ Physical activity derived from the Global Physical Activity Questionnaire (GPAQ) developed by the World Health Organization. A MET (metabolic equivalent) is defined at 1 kcal/kg/hour and is equivalent to the energy cost of sitting quietly. ^3^ Cardiovascular Disease Risk based on the Framingham Study criterion: a composite of CHD (coronary death, myocardial infarction, coronary insufficiency, and angina), cerebrovascular events (including ischemic stroke, hemorrhagic stroke, and transient ischemic attack), peripheral artery disease (intermittent claudication), and heart failure. ^4^ Hypertension defined by NHANES definition: BP ≥ 140/90 mm/Hg or if the participant self-reported as currently taking antihypertensive medications. Participants without a blood pressure measurement and with no medication use were assumed to be not hypertensive. ^5^ Type 2 diabetes based on American Diabetes Association definition of fasting blood glucose ≥ 126 mg/dL or post-oral glucose tolerance test ≥ 200 mg/dL or HbA1C ≥ 6.5% or taking glucose-lowering medication.

**Table 2:** Baseline dietary intake by quintile of dairy servings ^1^, HCHS/SOL.

Dietary Variable ^2^	Q1 *n* = 2123	Q2 *n* = 2124	Q3 *n* = 2124	Q4 *n* = 2124	Q5 *n* = 2123	Overall *n* = 10,618
Total Energy, kcal/d	2040 (2015, 2066)	1958 (1936, 1980)	1945 (1916, 1974)	1896 (1868, 1923)	1928 (1900, 1955)	1871 (1856, 1886)
Total Dairy, s/d	2.40 (2.39, 2.42)	2.70 (2.69, 2.71)	2.95 (2.94, 2.96)	3.21 (3.19, 3.22)	3.72 (3.70, 3.74)	3.02 (3.00, 3.04)
Total Whole and Reduced Fat Dairy, s/d	2.26 (2.24, 2.27)	2.52 (2.51, 2.53)	2.75 (2.74, 2.77)	2.98 (2.96, 3.00)	3.45 (3.42, 3.47)	2.80 (2.79, 2.82)
Total Nonfat Dairy, s/d	0.15 (0.15, 0.15)	0.17 (0.17, 0.18)	0.20 (0.19, 0.20)	0.23 (0.22, 0.24)	0.27 (0.26, 0.29)	0.22 (0.21, 0.22)
Total Milk, s/d	0.54 (0.53, 0.55)	0.65 (0.63, 0.66)	0.76 (0.74, 0.77)	0.86 (0.84, 0.88)	1.10 (1.07, 1.13)	0.82 (0.81, 0.83)
Total Cheese, s/d	0.48 (0.47, 0.49)	0.51 (0.51, 0.52)	0.53 (0.53, 0.54)	0.54 (0.53, 0.55)	0.57 (0.56, 0.58)	0.50 (0.50, 0.51)
Total Yogurt, s/d	0.08 (0.08, 0.09)	0.09 (0.09, 0.09)	0.09 (0.09, 0.10)	0.09 (0.09, 0.10)	0.10 (0.10, 0.11)	0.10 (0.09, 0.10)
Total Cream, s/d	0.83 (0.82, 0.84)	0.86 (0.85, 0.87)	0.90 (0.89, 0.91)	0.94 (0.93, 0.95)	1.02 (1.00, 1.03)	0.89 (0.89, 0.90)
Total Butter, s/d	0.48 (0.46, 0.49)	0.59 (0.58, 0.60)	0.67 (0.66, 0.68)	0.76 (0.75, 0.78)	0.93 (0.91, 0.95)	0.71 (0.70, 0.72)
Added Sugar, g/d	67.95 (66.24, 69.65)	66.16 (64.56, 67.76)	65.17 (63.47, 66.87)	64.86 (63.32, 66.40)	68.89 (67.50, 70.27)	62.34 (61.64, 63.03)
Omega 3 Fatty Acids, mg/d	80.07 (78.07, 82.07)	85.26 (83.19, 87.33)	88.27 (85.79, 90.75)	88.77 (86.69, 90.85)	89.20 (87.04, 91.35)	84.82 (83.62, 86.01)
Vegetables, s/d	2.12 (2.08, 2.16)	2.10 (2.06, 2.14)	2.05 (2.00, 2.10)	1.96 (1.92, 2.01)	1.79 (1.74, 1.84)	2.00 (1.97, 2.03)
Whole Fruit, s/d	1.12 (1.08, 1.16)	1.12 (1.08, 1.16)	1.12 (1.07, 1.17)	1.11 (1.06, 1.15)	0.99 (0.94, 1.04)	1.15 (1.12, 1.18)
Whole Grains, s/d	2.12 (1.96, 2.28)	1.79 (1.67, 1.91)	1.62 (1.51, 1.72)	1.40 (1.32, 1.49)	1.25 (1.16, 1.34)	1.53 (1.46, 1.61)

^1^ Data are mean (95% CI) or proportion values by dairy consumption category. Participants were categorized by quintile of total dairy intake after adjusting dairy intake for total energy using the residual method. ^2^ National Cancer Institute method used to estimate predicted servings, grams or kcal based on two 24 h recalls collected at baseline.

**Table 3:** Prospective associations between baseline dairy consumption and 6-year CESD10 score among Hispanic/Latino adults, HCHS/SOL (*n* = 10,618).

Exposure	Model ^1^	β (95% CI) ^2^	Standardized β (95% CI) ^3^	*p*-Value
Total Dairy (s/d)	1	−0.19 (−0.52, 0.13)	−0.017 (−0.047, 0.012)	0.25
	2	−0.21 (−0.53, 0.12)	−0.019 (−0.048, 0.011)	0.22
Full- and Reduced-Fat Dairy (s/d)	1	−0.15 (−0.50, 0.19)	−0.013 (−0.043, 0.017)	0.39
	2	−0.17 (−0.52, 0.17)	−0.015 (−0.045, 0.015)	0.32
Nonfat Dairy (s/d)	1	−0.40 (−1.33, 0.52)	−0.011 (−0.035, 0.014)	0.39
	2	−0.35 (−1.27, 0.57)	−0.009 (−0.033, 0.015)	0.46

Total dairy was calculated as the sum of servings per day (s/d) from milk, cheese, yogurt, cream and butter. Full- and reduced-fat dairy included whole- and reduced-fat milk, cheese, yogurt, cream and butter. ^1^ Model 1 covariates included age, sex, field center, Hispanic/Latino background, education level, income level, acculturation, smoking status, baseline CESD10 score, lapsed time between tests in days, BMI, physical activity level, use of antidepressants, history of CVD, hypertension, T2D, and total energy. Model 2 covariates included Model 1 covariates plus added sugar (g/d), omega-3 fatty acids (mg/d), whole grains (s/d), fruits (s/d), and vegetables (s/d). ^2^ Values represent the difference in 6-year CESD10 scores per additional daily serving of dairy consumed at baseline. ^3^ Values are interpreted as the standard deviation difference in 6-year CESD10 scores per one standard deviation increase in daily dairy servings consumed at baseline.

**Table 4:** Prospective associations between baseline dairy product consumption and 6-year CESD10 score among Hispanic/Latino adults, HCHS/SOL (*n* = 10,618).

Exposure	Model ^1^	β (95% CI) ^2^	Standardized β (95% CI) ^3^	*p*-Value
Milk (s/d)	1	−0.06 (−0.47, 0.34)	−0.004 (−0.027, 0.020)	0.77
	2	−0.10 (−0.50, 0.30)	−0.006 (−0.029, 0.018)	0.63
Cheese (s/d)	1	1.66 (−0.33, 3.64)	0.037 (−0.007, 0.081)	0.10
	2	1.71 (−0.26, 3.67)	0.038 (−0.006, 0.081)	0.09
Yogurt (s/d)	1	−4.29 (−6.92, −1.67)	−0.037 (−0.059, −0.014)	0.001
	2	−4.20 (−6.83, −1.56)	−0.036 (−0.058, −0.013)	0.002
Cream (s/d)	1	−0.18 (−1.28, 0.93)	−0.005 (−0.038, 0.027)	0.76
	2	−0.16 (−1.26, 0.95)	−0.005 (−0.037, 0.028)	0.78
Butter (s/d)	1	−1.12 (−2.06, −0.17)	−0.051 (−0.093, −0.008)	0.021
	2	−1.08 (−2.04, −0.12)	−0.049 (−0.092, −0.006)	0.027

^1^ Model 1 covariates included age, sex, field center, Hispanic/Latino background, education level, income level, acculturation, smoking status, baseline CESD10 score, lapsed time between tests in days, BMI, physical activity level, use of antidepressants, history of CVD, hypertension, T2D, and total energy. Model 2 covariates included Model 1 covariates plus added sugar (g/d), omega-3 fatty acids (mg/d), whole grains (s/d), fruits (s/d), and vegetables (s/d). ^2^ Values represent the difference in 6-year CESD10 scores per additional daily serving of dairy consumed at baseline. ^3^ Values are interpreted as the standard deviation difference in 6-year CESD10 scores per one standard deviation increase in daily dairy servings consumed at baseline.

## Data Availability

Restrictions apply to the availability of these data. Researchers can learn more about how to access HCHS/SOL resources through the study at https://www.nhlbi.nih.gov/science/hispanic-community-health-studystudy-latinos-hchssol (accessed on 22 October 2022) and through NHLBI’s cloud-based ecosystem, Biologic Specimen and Data Repository Information Coordinating Center (BioLINCC/BioData Catalyst) (https://biolincc.nhlbi.nih.gov/home/) (accessed on 22 October 2022).

## Abbreviations

The following abbreviations are used in this manuscript:

BMI	Body Mass Index
CESD10	10-Item Center for Epidemiological Studies Depression Scale
CVD	Cardiovascular Disease
FFQ	Food Frequency Questionnaire
GPAQ	Global Physical Activity Questionnaire
HCHS/SOL	Hispanic Community Health Study/Study of Latinos
MET	Metabolic Equivalent
NCC	Nutrition Coordinating Center
NCI	National Cancer Institute
NDSR	Nutrition Data System for Research
SCFA	Short-Chain Fatty Acid
T2D	Type 2 Diabetes

## Appendix A

Supplemental tables for additional sensitivity analyses shown below:

**Table A1:** Prospective associations between baseline milk, cheese and yogurt consumption and 6-year CESD10 score among Hispanic/Latino adults, HCHS/SOL (*n* = 10,618).

Exposure	Model ^1^	β (95% CI) ^2^	Standardized β (95% CI) ^3^	*p*-Value
Total Milk, Cheese, and Yogurt (s/d)	1	−0.05 (−0.44, 0.34)	−0.003 (−0.028, 0.022)	0.81
	2	−0.08 (−0.48, 0.31)	−0.005 (−0.030, 0.020)	0.69
Total Whole and Reduced Fat Milk, Cheese, and Yogurt (s/d)	1	0.04 (−0.39, 0.46)	0.002 (−0.023, 0.027)	0.86
	2	−0.01 (−0.44, 0.41)	−0.001 (−0.026, 0.025)	0.95
Total Nonfat Milk, Cheese, and Yogurt (s/d)	1	−0.40 (−1.33, 0.52)	−0.011 (−0.035, 0.014)	0.39
	2	−0.35 (−1.27, 0.57)	−0.009 (−0.033, 0.015)	0.46

^1^ Model 1 covariates included age, sex, field center, Hispanic/Latino background, education level, income level, acculturation, smoking status, baseline CESD10 score, lapsed time between tests in days, BMI, physical activity level, use of antidepressants, history of CVD, hypertension, T2D, and total energy. Model 2 covariates included Model 1 covariates plus added sugar (g/d), omega-3 fatty acids (mg/d), whole grains (s/d), fruits (s/d), and vegetables (s/d). ^2^ Values represent the difference in 6-year CESD10 scores per additional daily serving of dairy consumed at baseline. ^3^ Values are interpreted as the standard deviation difference in 6-year CESD10 scores per one standard deviation increase in daily dairy servings consumed at baseline.

**Table A2:** Prospective associations between baseline dairy consumption and 6-year CESD10 score among Hispanic/Latino adults not taking antidepressant medication, HCHS/SOL (*n* = 10,102).

Exposure	Model ^1^	β (95% CI) ^2^	Standardized β (95% CI) ^3^	*p*-Value
Total Dairy (s/d)	1	−0.23 (−0.56, 0.10)	−0.021 (−0.050, 0.009)	0.18
	2	−0.24 (−0.57, 0.09)	−0.022 (−0.052, 0.008)	0.15
Full- and Reduced-Fat Dairy (s/d)	1	−0.21 (−0.56, 0.15)	−0.018 (−0.048, 0.013)	0.25
	2	−0.23 (−0.58, 0.12)	−0.020 (−0.050, 0.011)	0.21
Fat-Free Dairy (s/d)	1	−0.30 (−1.31, 0.71)	−0.008 (−0.034, 0.018)	0.56
	2	−0.25 (−1.26, 0.75)	−0.007 (−0.033, 0.020)	0.62

^1^ Model 1 covariates included age, sex, field center, Hispanic/Latino background, education level, income level, acculturation, smoking status, baseline CESD10 score, lapsed time between tests in days, BMI, physical activity level, history of CVD, hypertension, T2D, and total energy. Model 2 covariates included Model 1 covariates plus added sugar (g/d), omega-3 fatty acids (mg/d), whole grains (s/d), fruits (s/d), and vegetables (s/d). ^2^ Values represent the difference in 6-year CESD10 scores per additional daily serving of dairy consumed at baseline. ^3^ Values are interpreted as the standard deviation difference in 6-year CESD10 scores per one standard deviation increase in daily dairy servings consumed at baseline.
